# Editorial: Tropism, Mapping, Modeling, or Therapy Using Canine Adenovirus Type 2 (CAV-2) Vectors in the CNS

**DOI:** 10.3389/fnmol.2021.636476

**Published:** 2021-02-11

**Authors:** Iria Gonzalez Dopeso-Reyes, Mathieu Wolff, Melissa R. Andrews, Eric J. Kremer

**Affiliations:** ^1^Institut de Génétique Moléculaire de Montpellier, Université de Montpellier, CNRS, Montpellier, France; ^2^Unité Mixte de Recherche 5287, Centre National de la Recherche Scientifique, INCIA, Université de Bordeaux, Bordeaux, France; ^3^School of Biological Sciences, University of Southampton, Southampton, United Kingdom

**Keywords:** CAV-2, neural circuit tracing, gene transfer, Parkinson's disease, coxsackievirus adenovirus receptor, retrograde transport, DREADDs, optogenetics

This Frontiers in Molecular Neuroscience Research Topic addresses tropism, mapping, modeling, and therapy using canine adenovirus (CAV-2) vectors in the CNS. Genetic modification of cells in the brain or spinal cord has undergone a revolution in the last decade, both in terms of furthering our understanding of brain structure/function and for therapeutic reasons. The current collection of viral vectors gives scientists a multifunctional kit to target neurons in many brain structures. Approximately 20 years ago, we demonstrated the capacity of CAV-2 vectors to preferentially infect neurons and their high retrograde transport capacity (Soudais et al., 2001). Richard Palmiter's lab was one of the first to take advantage of CAV-2 vector retrograde transport and addressed the physiological functions of the substantia nigra (Hnasko et al., 2005, 2006). The evolution of gene transfer vectors for the CNS has not abated. In this *Research Topic*, our goal was to provide a readily accessible collection of reviews and primary studies that epitomize the advantages and drawbacks of CAV-2 vectors in the mammalian CNS.

For some neuroscientists, the idiosyncrasies of each vector platform are confusing because the best choice will depend upon their unique question(s). The first question is likely “*Which vector is best to infect neurons?*” While adenoviral vectors can use numerous cell surface molecules to eventually infect cells (Arnberg, 2012), most of the >200 types have not been tested in the CNS. The uniqueness with CAV-2 is that for all intents and purposes it depends only on a cell adhesion molecule called “coxsackievirus and adenovirus receptor” (CAR) (Soudais et al., 2001, Salinas et al., 2009). Fortunately, CAR is highly conserved, and all mammals appear to express CAR on neurons in the brain parenchyma (Wehbi et al.). CAV-2 vectors have been used efficiently in rodents (Morceau et al.; Martel et al.; Cerpa et al.; Kakava-Georgiadou et al.), dogs (Cubizolle et al., 2014), and non-human primates (NHP) (di Caudo et al., reviewed by Lasbleiz et al.). Not only are CAV-2 vectors efficient but they also appear to drive highly selective expression, which is sometimes a concern with AAV-based conditional constructs with leakage of gene expression, prompting the need to either work out the optimal virus titration (Morceau et al.) or rely on CAV-2 vectors and transgenic lines (Cerpa et al.).

The second question is likely to be— “*Which vector has the capacity to harbor my expression cassette of interest?*” E1/E3-deleted CAV-2 vectors have a cloning capacity >7 kbp, while helper-dependent CAV-2 vectors (HD-CAV2) have a cloning capacity up to 36 kbp (del Rio et al.). This characteristic sets CAV-2 vectors apart from others (except HSV vectors, which have a theoretical capacity of 150 kb). By exploiting this characteristic, di Caudo et al. and Mestre-Francés et al. (2019) created vectors harboring a leucine-rich repeat kinase 2 (LRRK2) cassette containing a point mutation (G2019S) to produce genetic NHP models of Parkinson's disease (reviewed by Lasbleiz et al.).

The third issue typically concerns the type of neuron that needs to be targeted. CAR is present mainly but not exclusively in the axons (Wehbi et al.) of different neuronal populations (reviewed by Lavoie and Liu), allowing one to target different types of neurons in different areas of the CNS in rodents and the peripheral nervous system in primates (Bohlen et al.). If one combines the presence of CAR and a specific promoter, transgene expression can be targeted to cholinergic interneurons (Martel et al.) or neurons in the locus coeruleus (Hirschberg et al., 2017; Xiang et al., 2019; Hayat et al., 2020). Furthermore, Lavoie and Liu have compiled a list of possible neuronal subtypes (glutamatergic, dopaminergic, catecholaminergic, serotoninergic, oxytonic, and GABAergic) that have been effectively transduced by CAV-2 vectors and they also point out that the lack of infection of some projecting neuron types is possible.

The ability of the neuronal pre-synapse to endocytose CAV-2 particles and confuse them for cargo destined for transport to the soma (retrograde transport) has provided neuroscientists with one of the first efficient vectors to address neuronal circuitry. How the 100 billion neurons, via the trillions of synapses, in a human brain interact, and how a given subset influences behavior may never be understood. However, this daunting task has not stopped many from trying to use less complex systems to create a rough blueprint as a starting point. In addition to circuitry, the advent of opto- and chemogenetics now allows one to address the function of selected neuronal populations (Cerpa et al.).

Finally, CAV-2 vectors (both E1/E3-deleted and HD) are also capable of long-term expression (del Rio et al.; Hirschberg et al., 2017). A study of the transcriptional pathways affected by HD-CAV2 demonstrated that there was a modest modulation of genes involved in the immune response, intracellular trafficking, and transcriptional regulation (reviewed by del Rio et al.).

In summary, the future of neuron-targeted gene transfer has many options—and in particular, CAV-2 vectors have many favorable characteristics.

## Author Contributions

IGD-R, MW, MA, and EK wrote the manuscript, edited, and approved of the manuscript.

## Conflict of Interest

The authors declare that the research was conducted in the absence of any commercial or financial relationships that could be construed as a potential conflict of interest.
